# Unlocking the Potential: Semaglutide’s Impact on Alzheimer’s and Parkinson’s Disease in Animal Models

**DOI:** 10.3390/cimb46060354

**Published:** 2024-06-13

**Authors:** Andreea Daniela Meca, Ianis Kevyn Stefan Boboc, Liliana Mititelu-Tartau, Maria Bogdan

**Affiliations:** 1 Department of Pharmacology, Faculty of Pharmacy, University of Medicine and Pharmacy, 200349 Craiova, Romania; andreea_mdc@yahoo.com (A.D.M.); kevynboboc@gmail.com (I.K.S.B.); 2 Department of Pharmacology, Faculty of Medicine, ‘Grigore T. Popa’ University of Medicine and Pharmacy, 700115 Iasi, Romania

**Keywords:** semaglutide, neuroprotective, Alzheimer’s disease, Parkinson’s disease, animal models

## Abstract

Semaglutide (SEM), a glucagon-like peptide-1 receptor agonist, has garnered increasing interest for its potential therapeutic effects in neurodegenerative disorders such as Alzheimer’s disease (AD) and Parkinson’s disease (PD). This review provides a comprehensive description of SEM’s mechanism of action and its effects in preclinical studies of these debilitating conditions. In animal models of AD, SEM has proved beneficial effects on multiple pathological hallmarks of the disease. SEM administration has been associated with reductions in amyloid-beta plaque deposition and mitigation of neuroinflammation. Moreover, SEM treatment has been shown to ameliorate behavioral deficits related to anxiety and social interaction. SEM-treated animals exhibit improvements in spatial learning and memory retention tasks, as evidenced by enhanced performance in maze navigation tests and novel object recognition assays. Similarly, in animal models of PD, SEM has demonstrated promising neuroprotective effects through various mechanisms. These include modulation of neuroinflammation, enhancement of mitochondrial function, and promotion of neurogenesis. Additionally, SEM has been shown to improve motor function and ameliorate dopaminergic neuronal loss, offering the potential for disease-modifying treatment strategies. Overall, the accumulating evidence from preclinical studies suggests that SEM holds promise as a novel therapeutic approach for AD and PD. Further research is warranted to elucidate the underlying mechanisms of SEM’s neuroprotective effects and to translate these findings into clinical applications for the treatment of these devastating neurodegenerative disorders.

## 1. Introduction

Alzheimer’s disease (AD) is a neurodegenerative condition marked by cognitive decline, memory impairment, and eventual loss of executive function [1,2]. It accounts for 50% to 75% of diagnosed dementia cases [3]. AD is classified into two types: senile (sporadic), which is predominant in 95–98% of cases, and presenile (familial) [4,5]. It is hypothesized that the neurodegenerative processes associated with sporadic AD may begin as early as 20 years before the onset of clinical symptoms [6].

Parkinson’s disease (PD) is a prevalent condition among the elderly, characterized by progressive neurodegeneration that significantly impacts patients’ lives, particularly when left unmanaged [7,8]. Typically initiating around the ages of 60–65, though occasionally manifesting earlier, it ranks among the most prevalent movement disorders [9,10,11,12]. Primarily triggered by dopamine deficiency, PD chiefly involves the degeneration of neurons responsible for dopamine production within the substantia nigra [10,11,12,13,14].

Various analogs of glucagon-like peptide-1 (GLP-1) have been examined for their potential in AD and PD, including those that activate receptors for a single type of incretin [single incretin receptor agonists—exenatide, liraglutide, lixisenatide, and semaglutide (SEM)] and those that activate receptors for both types of incretins (dual incretin receptor agonists—Peptide 19, DA4-JC, DA5-CH, DA3-CH) [12,13,14,15,16].

**Figure 1:**
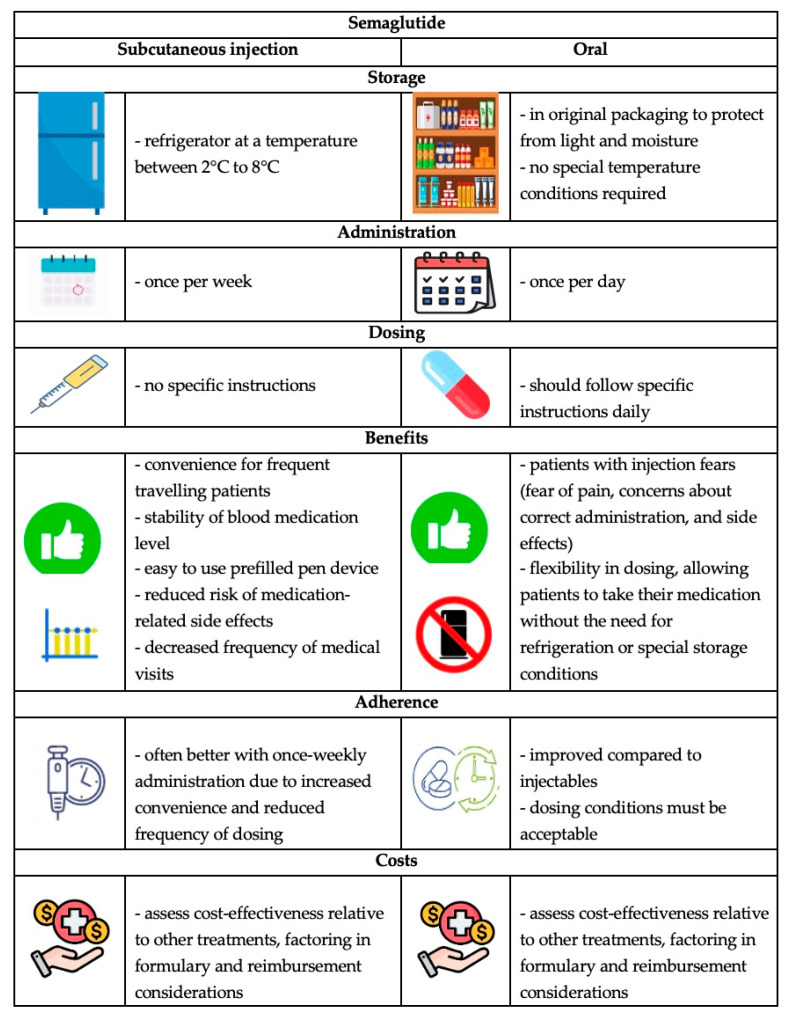
Characteristics of subcutaneous and oral formulations of SEM.

SEM, a long-acting peptide-modified human GLP-1 receptor agonist resistant to protease, has been shown to significantly reduce blood glucose and glycated hemoglobin (HbA1c) levels compared to short-acting agents [16,17,18,19,20]. It was approved for type 2 diabetes in the USA in 2017 and in Canada and Europe in 2018 for 1 mg SEM administered subcutaneously once weekly, exhibiting a safety profile similar to other antidiabetic agents and offering additional advantages [21,22]. In 2019 in the USA and 2020 in Europe, an oral dose of a maximum of 14 mg daily of SEM received approval as type 2 diabetes pharmacotherapy [10,21,22] so that doctors can choose the most suitable formulation for the patients’ necessities (Figure 1) [10].

Nevertheless, in 2021, the FDA offered an additional approval for 2.4 mg of SEM, administered once a week subcutaneously, as an adjunct able to reduce caloric intake in individuals diagnosed with obesity (BMI higher than 30 kg/m^2^ or higher than 27 kg/m^2^ with at least one comorbidity related to weight) [10,21,22]. Due to its pharmacokinetic profile, SEM presents a slightly different toxicity profile when compared to other GLP-1 receptor agonists, such as less frequent injection site effects and hypoglycemic risk (that can further increase when associated with insulin or sulfonylureas), but also more frequent vomiting [9].

SEM boasts an extended half-life of 7 days in the bloodstream, allowing for once-weekly administration at any time of the day, with or without food. The initial injectable dose typically starts at 0.25 mg, while the regular dosage ranges from 0.5 to 1 mg [20]. Additionally, it is available in oral tablet form in strengths of 3, 7, or 14 mg, exhibiting similar efficacy to subcutaneous doses. SEM stands out as the sole GLP-1 receptor agonist offered in both injectable and oral pharmaceutical formulations [9,14,23]. With a 94% homology to native GLP-1, SEM results in only 1% presence of antibodies against SEM or GLP-1 post-treatment, thus minimizing the risk of allergic reactions [23,24]. Furthermore, it can be excreted via both urine and feces, eliminating the need for dose adjustment in cases of renal insufficiency [25,26]. An absorption enhancer (SNAC or sodium N-[8-(2-hydroxybenzoyl)]-aminocaprylate) for SEM has been noticed due to its ability to protect this GLP-1 agonist from proteolytic degradation within the stomach and also to facilitate active intestinal transport [9].

The objective of this review is to offer an extensive description of SEM’s involvement in the pathophysiology of AD and PD, emphasizing the drug’s mechanism of action and its relevance in animal models of these disorders.

## 2. SEM Mechanism of Action

GLP-1 receptor agonists, also referred to as incretin analogs, play a crucial role in maintaining glycemic control by stimulating natural mechanisms and responses to nutrient intake [25]. This process involves the release of the incretin hormone GLP-1 from intestinal L cells following meals [26,27,28]. GLP-1 receptor agonists bind to specific receptors distributed widely throughout the body, including pancreatic islets, renal, pulmonary, cardiovascular, gastrointestinal, immune, and brain cells [19]. GLP-1 and GIP (glucose-dependent insulinotropic polypeptide) receptors belong to the G protein-coupled B1 class receptors (the secretin family), which can trigger the production of cAMP (cyclic adenosine monophosphate) and undergo conformational changes upon activation [27]. The activation of GLP-1 and GIP hormones consequently leads to delayed gastric emptying and modulation of insulin secretion [24,25]. However, shortly after their release, GLP-1 and GIP are broken down by the enzyme dipeptidyl peptidase-4 (DPP-4), resulting in their short half-lives as endogenous molecules [27,28,29].

As versatile peptides, GLP-1 receptor agonists, such as SEM, play a role in enhancing insulin and amylin secretion by stimulating pancreatic β-cells while simultaneously inhibiting glucagon secretion from pancreatic α-cells in a glucose-dependent manner. They also decrease hepatic glucose production, increase feelings of satiety, and regulate appetite through central mechanisms [19,29,30]. Additionally, GLP-1 is involved in promoting pancreatic β-cell proliferation and preventing apoptosis [25]. SEM promotes insulin uptake in peripheral tissues, resulting in weight loss, reduced risk of hepatic steatosis, and increased lipolysis, making them beneficial for patients with type 2 diabetes and obesity [19,28]. Activation of GLP-1 receptors correlates with increased intracellular calcium levels and ERK1/2 phosphorylation to stimulate insulin production [25,28]. Furthermore, SEM can enhance glycolysis by upregulating GLUT2 transporters and glucokinase expression. SEM also regulates tissue sensitivity to insulin [26].

These benefits arise from the activation of peripheral and central intestinal and brain receptors, particularly those located in hypothalamic satiety centers, which are also involved in regulating perceptions of motivation and reward-related to food intake [19,23]. Acting as a multifunctional peptide, brain-derived GLP-1 is synthesized by pre-proglucagon neurons situated in the caudal area of the nucleus tractus solitarius (NTS) [30]. GLP-1 receptors are expressed in various regions of the brain, including the frontal cortex, hippocampus, substantia nigra, and cerebellum [24]. Moreover, GLP-1 has the capacity to modulate diverse brain functions such as water intake, energy homeostasis, thermogenesis, neural genesis, degeneration, and responses to stress [31]. The amelioration of cognitive impairment has led to the recommendation of GLP-1 receptor agonists for neurological deficits associated with type 2 diabetes and obesity [28]. Additionally, most GLP-1 receptor agonists can penetrate the blood-brain barrier (BBB) and initiate neuroprotective effects, as shown in a murine study for the i.v. injection of 1 × 10^6^ counts per minute of radioactively labeled exenatide, lixisenatide, Peptide 17, Peptide 21, DA3-CH, DA4-JC [18,32].

GLP-1 receptor agonists are distinguished by their prolonged half-life compared to endogenous GLP-1, allowing them to remain bound to specific receptors for an extended period [31]. Their safety profile includes a low risk of hypoglycemia, facilitation of weight loss, beneficial cardiovascular and renal effects, reduced dosing frequency, and decreased injection burden, leading to improved treatment adherence among patients [19,20]. Additionally, one of the most significant advantages of GLP-1 receptor agonists is their ability to traverse the BBB to varying extents within the class [9,10]. Even when compared to other long-acting agents, such as exenatide with extended release and dulaglutide, subcutaneous SEM has demonstrated superior efficacy in improving glycemic control [28]. Furthermore, SEM exhibits pleiotropic effects by correcting insulin resistance, protecting against glucolipotoxicity, and inhibiting apoptosis [14,28]. This GLP-1 receptor agonist can also enhance lipid profiles by activating an intrinsic gut–liver signaling pathway, thereby reducing VLDL production [14]. Moreover, SEM has been shown to lower systolic blood pressure, protect renal function, improve insulin resistance, and induce anti-obesity effects [14,32]. However, recent research has focused on investigating the potential of SEM to provide neuroprotection and mitigate the progression of various neurodegenerative diseases. In this review, we examine the therapeutic potential of SEM and its underlying neuroprotective pathways in both AD and PD.

## 3. Therapeutic Potential of SEM in Neurodegenerative Diseases

Impaired cognitive function is associated with insulin resistance, as neuronal metabolism and synaptic efficacy suffer from the desensitization of insulin signaling pathways [33,34]. Insulin is crucial in activating various growth factors that further stimulate brain receptors, thereby regulating mitochondrial functionality, cellular energy utilization, cell survival, and synaptic processes [33,35]. Reduced or delayed neuronal repair due to impaired insulin signaling has been correlated with an increased risk of developing neurodegenerative pathologies [33,35]. Since neurons cannot produce or store glucose, they rely on the activation and sensitization of specific proteins acting as glucose transporters to prevent neurodegeneration [35]. GLP-1 receptor agonists, more specifically SEM, can compensate for deficiencies in insulin signaling by activating the same growth factor cascades as insulin [33,34]. Moreover, recent research has unveiled the neurotrophic and neuroprotective activities of GLP-1 [36,37].

Neuronal loss in AD has been associated with neurocyte apoptosis [36,38]. However, both insulin resistance and hyperinsulinemia can contribute to cognitive impairment in adults, highlighting impaired tissue sensitivity to insulin as a pathogenic mechanism in AD [29]. As demonstrated by Yu et al. in their experimental study using the water navigation task, liraglutide has shown efficacy in preventing neuronal apoptosis [38]. GLP-1 receptor activation can also mitigate tau phosphorylation and amyloid β deposition, critical factors in delaying the progression of AD [29,30]. GLP-1 receptor agonists (especially SEM) have been found to reduce neuronal apoptosis, neuroinflammation, synapse loss, and brain accumulation of advanced glycation end products, as concluded by a systematic review [25].

Moreover, the onset of PD has been linked to type 2 diabetes and desensitized insulin signaling [23,34,39]. In various mouse models of PD, GLP-1 mimetics have shown efficacy in reducing chronic neuronal inflammation, dopaminergic neuron loss, oxidative stress, and α-synuclein levels, ultimately leading to improved motor coordination [23,34]. Activation of GLP-1 brain receptors has been found to mitigate microglial neurotoxicity by inhibiting A1 astrocyte activity, thereby delaying neuronal death in neurodegenerative disorders [29]. Notably, liraglutide and lixisenatide demonstrated superior efficacy compared to other short-acting agents in maintaining tyrosine hydroxylase expression, a crucial enzyme in dopamine production [34]. In experimental mouse models, exenatide restored motor functionality, while SEM prevented the loss of dopaminergic neurons by down-regulating microgliosis and astrogliosis, thus reducing chronic inflammation [20,23,25]. Furthermore, SEM normalized the expression of autophagy-related proteins in the substantia nigra and striatum in mice, including P62, ATG7, Beclin, and LC3, leading to the alleviation of oxidative stress and promotion of neurovascular reconstruction [31,32,39]. When compared in equivalent doses in experimental PD models, SEM exhibited superior efficacy in restoring mitochondrial functionality and antioxidative balance by enhancing autophagy [23,39].

SEM has been shown to reduce oxidative stress, a critical factor in NMDA excitotoxicity. By lowering oxidative stress levels, SEM can help mitigate the damage caused by excessive Ca^2+^ influx and reactive oxygen species (ROS) generation [40]. This reduction in oxidative stress also helps preserve the integrity and function of cholinergic neurons, thereby supporting acetylcholine production [41,42].

SEM has been found to improve mitochondrial function, which could help protect neurons from excitotoxic damage. Enhancing mitochondrial efficiency is crucial for maintaining neuronal health and preventing the cascade of events leading to cell death in AD [23,43].

By modulating the release and uptake of glutamate, SEM could help prevent the overactivation of NMDA receptors, thereby reducing excitotoxicity. This regulation of glutamate dynamics is essential for protecting neurons from the harmful effects of excessive glutamate signaling [44,45,46].

Chronic neuroinflammation is a hallmark of AD and is exacerbated by NMDA excitotoxicity. SEM exhibits anti-inflammatory properties, reducing the release of pro-inflammatory cytokines and potentially lessening the neuronal damage associated with excitotoxicity [44,47,48].

Although SEM’s primary mechanism is not the inhibition of acetylcholinesterase, there is some evidence to suggest that GLP-1 receptor agonists can reduce the activity of acetylcholinesterase, increasing the availability of acetylcholine in the brain, albeit this effect is less pronounced compared to traditional acetylcholinesterase inhibitors used in AD treatment. Furthermore, SEM can enhance the cholinergic system function by supporting the growth and maintenance of cholinergic synapses [41,45].

**Figure 2:**
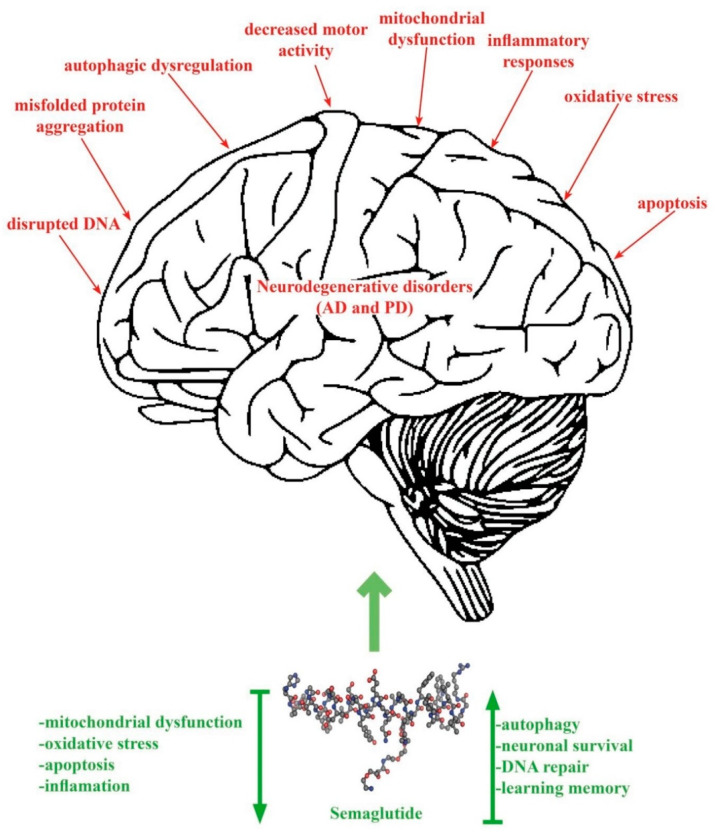
The pathogenesis of AD and PD and the effects of SEM.

Figure 2 shows the pathogenic mechanisms of AD and PD and the effects (decrease/increase) produced by SEM therapy.

Various experimental studies have shown the neurotrophic effects of GLP-1 agonists, although the exact mechanism of neuroprotection is not yet fully understood [18,20]. SEM, especially, is considered a versatile multifaceted agent [49,50]; it can activate both the production and signaling of neuronal stem cells [49].

Incretin signaling has also proven beneficial with regard to the outgrowth of the essential synaptic components called neurites through a mechanism similar to that of the nerve growth factor (NGF) [20,51]. Nevertheless, chronic administration of GLP-1 agonists (for more than 4 weeks) in mice models of diabetes has led to early DCX-positive neurons in the dentate gyrus area [51]. Neuronal precursors and other immature nervous cells usually express DCX, a microtubule-associated protein, suggesting an active division process and multiplication [51]. After binding and activating GLP-1 receptors, cAMP levels increase rapidly and activate two pathways through PI3K (phosphoinositide 3-kinase) and protein kinase A, being involved in protein and mitochondrial biogenesis while inhibiting neuronal apoptosis [20,51]. Therefore, GLP-1 agonists have been associated with neurotrophic effects.

The PI3K/Akt signaling pathway has been experimentally correlated with the neuroprotective properties of SEM due to enhanced mitochondrial functionality and reduction of neuro-apoptotic reactions [20]. Another GLP-1 agonist, exenatide, has initiated cellular stem proliferation through ciliary neurotrophic factor (CNTF), increased Ki-67 gene expression, and also induced microglial activation with reduction of astrogliosis [51], suggesting GLP-1 agonists utility in both AD and PD.

Even more, SEM PI3K/Akt pathway activation led to inhibition of a proinflammatory and neurodegenerative serine/threonine kinase (GSK3β or glycogen synthase kinase-3), noted in various nervous system diseases [52,53]. However, regulation of GSK3β by treatment GLP-1 agonists is complex and needs to be investigated in further experimental or clinical studies. Among its neuroprotective and neurotrophic effects, SEM has activated proteolytic and autophagic mechanisms that further led to the diminished accumulation of α-synuclein and therefore maintained an equilibrium among dopaminergic neurons in the substantia nigra in experimental-induced PD models [29,49]. This suggests a strong correlation between SEM treatment and improved mitochondrial functionality with reduced impairment of autophagic and lysosomal pathways, which needs to be monitored in clinical studies [20].

## 4. SEM Pharmacokinetics in Animal Models

SEM, an acylated GLP-1 analog, emerges as a distinctive contender among the insulin receptor agonists (IRAs) scrutinized by the Salameh team [18]. Unlike linear peptides such as exenatide and lixisenatide, SEM exhibits acylation, a characteristic it shares with liraglutide. Notably, the acylated dual agonist, Peptide 19, stands out for its significantly greater lipid solubility compared to its counterparts, including liraglutide and SEM. This heightened lipid solubility could potentially influence the pharmacokinetics and distribution of Peptide 19 in vivo. Moreover, SEM demonstrates remarkable stability, with less than 2% degradation in serum and less than 12% in the whole brain over the specified time frame of 60 min [18,54,55,56,57]. In another study [58], the stability of SEM was evaluated under four different storage conditions (i.e., 4 h at room temperature, 7 days at −70 °C, 24 h in the autosampler, and freeze/thaw cycles). It was found that SEM exhibited average stability ranging from 93.94% to 106.13% in plasma and 91.64% to 107.31% in brain tissue, with no significant deviations observed under all tested conditions [58]. Such robust stability positions SEM favorably when compared to other IRAs like DA3-CH, which experiences higher degradation rates, indicating that SEM remains stable for routine analysis. These structural and stability distinctions underscore SEM’s potential advantages in bioavailability and durability, contributing to its unique profile within the landscape of insulin receptor agonists [18,58,59,60].

Following intravenous administration, SEM plasma concentrations exhibited a multi-exponential decline, with an average half-life (T1/2) of 9.26 ± 1.06 h and an average clearance and distribution volume estimated at 0.21 mL/min/kg (0.10 L/kg) [58]. In a study on CD-1 mice conducted by Salameh’s team, the serum clearance of SEM labeled with mCi Na 125I was 2.5 h. Concerning subcutaneous administration, SEM plasma concentration increased gradually, reaching peak concentration between 3 and 12 h, and decreased with an average half-life of 7.22–8.99 h [18]. The absolute bioavailability of SEM in rats was reported to be 76.65–82.85%. However, in humans, the half-life after s.c. administration ranges between 165 and 183 h, with the highest bioavailability (89%) among all GLP-1 agonists [59,60,61,62].

Brain influx rates were analyzed to assess SEM’s ability to cross the BBB. It was observed that there was no significant correlation between blood-to-serum (B/S) ratios and exposure time, indicating a lack of transport. Additionally, SEM, along with other tested IRAs, exhibited varying capabilities in accessing the brain parenchyma. Within a 15 min timeframe after i.v. injection, the majority of DA4-JC, DA3-CH, exenatide, Peptide 17, and lixisenatide successfully entered the brain parenchyma. Conversely, Peptide 21 and liraglutide were predominantly sequestered in brain endothelial cells during the same period, indicating an obstacle to complete passage across the capillary wall. Notably, SEM and Peptide 19 encountered challenges in reliable detection within the brain parenchyma [18,58,63].

Despite the assertion that SEM generally does not penetrate the BBB, there is intriguing evidence of localized distribution of SEM within the brain, particularly in the hypothalamus [63,64]. Concentrations of SEM in the hypothalamus were notably higher than those observed in other brain regions, exhibiting an average concentration and blood-brain partition coefficient (Kp) that were 2.26- and 1.82-fold higher, respectively, compared to the total brain following both intravenous and subcutaneous injections [58].

This localized distribution in the hypothalamus is significant as it aligns with the proposed effects of SEM in the brain, such as neuroprotection. These effects are believed to be associated with the interaction of SEM with GLP-1 receptors in the brain [64,65,66,67,68]. This paradoxical finding underscores the complexity of SEM’s interaction with the brain and challenges the conventional understanding of its BBB permeability.

## 5. SEM Potential Mechanism of Action in AD

AD is a progressive neurodegenerative disorder characterized by a decline in cognitive function, memory impairment, and changes in behavior and personality. It is the most common cause of dementia among older adults. The pathological disturbances in AD are multifaceted and involve a combination of genetic, molecular, and cellular mechanisms. The primary pathological hallmarks of AD include amyloid-beta (Aβ) plaques, neurofibrillary tangles (NFTs), neuroinflammation, and synaptic and neuronal loss [69].

Aβ plaques are extracellular deposits of amyloid-beta peptides that accumulate in the brain. These peptides are derived from the amyloid precursor protein (APP), which is cleaved by the enzymes β-secretase and γ-secretase. The accumulation of Aβ is thought to result from an imbalance between production and clearance. The aggregation of Aβ peptides leads to the formation of soluble oligomers, which are neurotoxic, and eventually to the deposition of insoluble fibrils in plaques. The toxic oligomers disrupt synaptic function, impairing communication between neurons. They also induce oxidative stress and inflammatory responses, contributing to neuronal damage and cell death [70].

NFTs are intracellular aggregates of hyperphosphorylated tau protein. Tau is normally associated with microtubules, aiding in their stabilization and function in neuronal transport. In AD, tau becomes abnormally phosphorylated, leading to its detachment from microtubules and aggregation into paired helical filaments that form NFTs. The formation of NFTs disrupts the microtubule network, impairing axonal transport and leading to neuronal dysfunction and death. The spread of tau pathology follows a stereotypical pattern, starting in the entorhinal cortex and hippocampus before progressing to other brain regions [71].

Neuroinflammation is a significant component of AD’s pathology. It involves activating microglia and astrocytes, the brain’s resident immune cells, in response to Aβ plaques and NFTs. Activated microglia and astrocytes release pro-inflammatory cytokines and chemokines, contributing to a chronic inflammatory environment. This inflammation exacerbates neuronal injury and promotes further Aβ and tau pathology. Additionally, chronic neuroinflammation can disrupt the BBB, leading to further neuronal damage [72].

Synaptic loss and neuronal death are key features of AD, leading to the clinical symptoms of cognitive decline and memory impairment. The combined effects of Aβ toxicity, tau pathology, and neuroinflammation result in synaptic dysfunction and loss. Synapses are the primary sites of neural communication, and their loss directly correlates with cognitive deficits. Neuronal death occurs through various mechanisms, including apoptosis and necrosis, leading to brain atrophy [73].

Genetic factors play a significant role in AD, especially in early-onset cases. Mutations in genes such as APP, PSEN1, and PSEN2 are associated with familial forms of AD, leading to increased production of Aβ [74,75].

Cerebrovascular disease and impaired cerebral blood flow are increasingly recognized as contributing factors in AD. Vascular dysfunction can exacerbate Aβ deposition and tau pathology by impairing clearance mechanisms and promoting hypoxia and oxidative stress. This can further enhance neuroinflammation and neuronal damage [76].

Increased oxidative stress, resulting from an imbalance between the production of ROS and antioxidant defenses, is observed in AD. This leads to lipid peroxidation, protein oxidation, and DNA damage, contributing to neuronal injury. Impaired mitochondrial function is a hallmark of AD, leading to reduced energy production, increased ROS, and activation of apoptotic pathways [77,78].

### 5.1. Cell Viability

SEM has shown protective effects against the detrimental effects of Aβ25-35 on SH-SY5Y cells [35] and has also been found to activate the SIRT1/GLUT4 pathway [33,36,64,65,66,67,68,79,80].

### 5.2. Apoptosis Inhibition

SEM prevents Aβ25-35-induced apoptosis [39], as evidenced by elevated levels of the prosurvival BCl2 protein and reduced levels of the proapoptotic Bax protein. BCl2 is recognized for its anti-apoptotic function, whereas Bax promotes apoptosis [35]. This alteration in their equilibrium implies a protective effect against programmed cell death [33,35,36].

### 5.3. Enhanced Autophagy

Initially, Aβ25-35 inhibits autophagy, but this inhibition is reversed in the presence of SEM [24,32,35,39]. Autophagy is a cellular process involving the degradation and recycling of cellular components. The reversal is confirmed by alterations in the expression of key autophagy-related proteins: LC3II (a marker of autophagosome formation) increases; ATG7 (an essential autophagy-related protein) exhibits enhanced expression; P62 (a protein degraded during autophagy) decreases; Beclin1 (a regulator of autophagy initiation) shows increased expression [35,68,80].

### 5.4. SIRT1/GLUT4 Pathway

SEM enhances the expression of Sirtuin 1 (SIRT1), a protein involved in various cellular processes such as glucose metabolism, energy regulation, and cellular stress response, as well as the expression of GLUT4 (Glucose transporter 4), a pivotal protein facilitating glucose transport into cells. GLUT4 holds particular significance in glucose metabolism [33,36]. The interplay between SIRT1 and GLUT4, known as the SIRT1/GLUT4 pathway, appears to be crucial [33,36,68]. This pathway likely contributes to the regulation of glucose metabolism, potentially augmenting glucose uptake by cells, particularly in regions like the hippocampal CA3 region [33,36,68].

### 5.5. Improved Aβ and Tau Pathology

SEM appears to mitigate Aβ pathology in the hippocampal CA3 region, implying a potential therapeutic effect on the aggregation or build-up of Aβ, a hallmark of AD [33,36,81,82]. Tau pathology involves the accumulation of abnormal tau protein, resulting in neurofibrillary tangles within nerve cells [33,36]. Tau pathology is another prominent characteristic of AD. SEM also exhibits the ability to alleviate tau pathology in the same hippocampal CA3 region, suggesting a potential influence on the abnormal aggregation or alteration of tau proteins, thereby contributing to the amelioration of AD-related pathological changes [33,36,82,83,84,85,86].

### 5.6. Reduction of Neuroinflammation

Another potential mechanism proposed for SEM in the context of dementia, which may also be extrapolated to AD, involves neuroprotection through the reduction of neuroinflammation [84,85,87], particularly concerning microglial involvement in neurodegenerative diseases. SEM has been demonstrated to decrease markers of systemic inflammation in patients, and both liraglutide and SEM have exhibited anti-inflammatory effects by mitigating the development of atherosclerotic plaques in mouse models deficient in APOE and LDLr [84,85,87].

## 6. SEM Potential Mechanism of Action in PD

The pathological disturbances in PD are complex and involve a combination of genetic, molecular, and cellular mechanisms. The primary pathological hallmarks include the loss of dopaminergic neurons in the substantia nigra, the presence of Lewy bodies, neuroinflammation, oxidative stress, and mitochondrial dysfunction. The most prominent pathological feature of PD is the selective loss of dopaminergic neurons in the substantia nigra pars compacta. The degeneration of these neurons leads to a significant reduction in dopamine levels in the striatum, a critical brain region involved in regulating movement. The loss of dopamine disrupts the balance between the excitatory and inhibitory pathways in the basal ganglia, leading to the characteristic motor symptoms of PD [16].

Lewy bodies are intracytoplasmic inclusions composed primarily of α-synuclein, a presynaptic neuronal protein. The presence of Lewy bodies is a key histopathological hallmark of PD. α-Synuclein undergoes misfolding and aggregation, forming insoluble fibrils that are deposited as Lewy bodies within neurons. The aggregation of α-synuclein is believed to impair various cellular functions, including synaptic vesicle trafficking, mitochondrial function, and protein degradation pathways, contributing to neuronal death [88,89].

Neuroinflammation is a significant feature of PD, involving the activation of microglia and astrocytes, the brain’s resident immune cells. Activated microglia and astrocytes release pro-inflammatory cytokines and chemokines, creating a chronic inflammatory environment. This inflammation contributes to neuronal injury and exacerbates the progression of the disease. Neuroinflammation may be triggered by the presence of α-synuclein aggregates and other cellular debris resulting from neuronal degeneration.

Oxidative stress plays a crucial role in the pathogenesis of PD. It results from an imbalance between the production of ROS and the brain’s ability to detoxify these reactive intermediates. Dopaminergic neurons are especially vulnerable to oxidative stress due to dopamine metabolism, which generates ROS. Oxidative stress can damage cellular components, including lipids, proteins, and DNA, leading to neuronal dysfunction and death. The accumulation of oxidized dopamine and α-synuclein also contributes to the formation of Lewy bodies [90,91].

Mitochondrial dysfunction is a well-established feature of PD, affecting neurons’ energy metabolism and viability. Mitochondria are responsible for ATP production through oxidative phosphorylation. In PD, there is evidence of impaired mitochondrial function, including deficiencies in complex I activity of the electron transport chain. This leads to reduced ATP production, increased ROS generation, and the activation of apoptotic pathways, contributing to neuronal death [92,93].

Genetic factors contribute significantly to the pathogenesis of PD, especially in familial cases. Mutations in these genes are associated with autosomal recessive forms of PD. They are involved in mitochondrial quality control, and their dysfunction leads to impaired mitochondrial function and increased susceptibility to oxidative stress [94]. Another pathological feature in PD is impairment of the ubiquitin-proteasome system and autophagy-lysosome pathway. These pathways are essential for the clearance of damaged proteins and organelles. Dysfunction in these systems leads to the accumulation of misfolded proteins and cellular debris, contributing to neuronal death [95,96].

### 6.1. Inhibition of Cell Death and Neurodegeneration

Following the 6-OHDA treatment, there was a notable decrease in cell viability. Nonetheless, the administration of either SEM or liraglutide reversed this decline in cell viability. Notably, no discernible differences were observed between the effects of SEM and liraglutide treatments [23].

Treatment with SEM improved neurodegeneration, especially in the ventral posterior medial and lateral nuclei of the thalamus (VPM/VPL) in Pla2g6−/− mice [97].

Untreated PD mice exhibited significant downregulation of BClxL and BCl2. However, SEM treatment increased levels of both BClxL and BCl2, restoring them to levels found in wild-type controls [97]. Zhang et al. (2018) demonstrated that MPTP induces apoptosis by inhibiting mitochondrial enzyme complex I, and SEM partly reverses this process by modulating BCl2 and Bax levels (Table 1) [32]. Additionally, SEM interferes with mechanisms leading to the accumulation and aggregation of α-synuclein (α-syn), reducing α-syn expression and potentially preventing the spread of toxic forms of α-syn between neurons, thus promoting neuronal survival [24,31,35,97,98,99,100,101,102,103].

**Figure 3:**
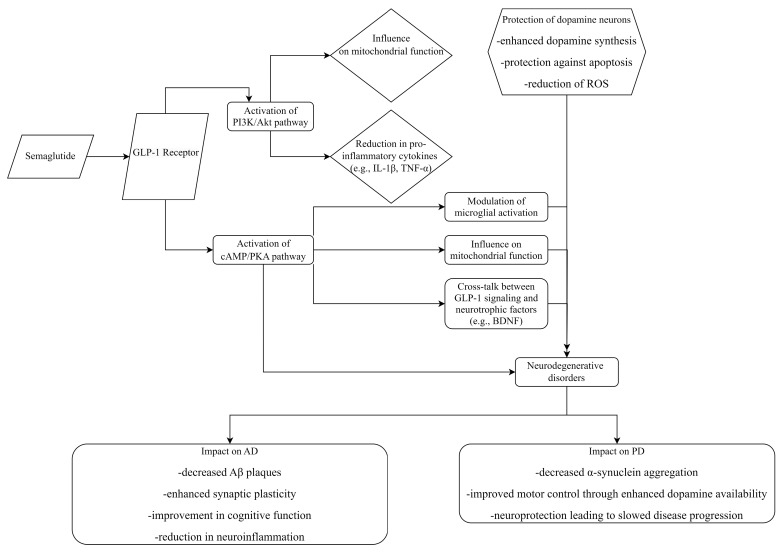
Mechanism of action of SEM and its impact on dopamine in AD and PD.

In the MPTP murine model of PD, which disrupts dopamine synthesis in the brain, both SEM and liraglutide restore dopamine synthesis. SEM proves more effective than liraglutide in normalizing TH expression [14,24,30,32], potentially by reducing the number of damaged dopaminergic neurons [30,32]. SEM’s efficacy in this context might be attributed to its ability to protect dopaminergic neurons against stress and damage rather than directly enhancing dopamine synthesis. In another murine PD model involving 6-hydroxydopamine (6-OHDA), SEM shields against 6-OHDA-induced damage to dopaminergic neurons in the substantia nigra [20,30]. Another potential mechanism involves restoring glial cell line-derived neurotrophic factor levels (GDNF), crucial for the survival of midbrain dopamine neurons implicated in PD [20,24,30,32,99,100,101]. Interestingly, SEM does not significantly impact the recovery of dopamine synthesis in the substantia nigra; however, the administration of DA5-CH exhibits higher dopamine levels [30,32,99,100,101]. This suggests that while SEM plays a critical role in maintaining the viability and function of dopaminergic neurons, its effects on dopamine synthesis might be indirect, focusing more on neuronal survival and health rather than directly increasing dopamine production (Figure 3).

SEM activates GLP-1 receptors, triggering cAMP/PKA and PI3K/Akt pathways, reducing oxidative stress and pro-inflammatory cytokines. This protects dopaminergic neurons therefore enhancing dopamine synthesis and preventing apoptosis. In PD, it improves motor control and slows neurodegeneration. In AD, it reduces beta-amyloid plaques and enhances cognitive function.

### 6.2. GSK3β Levels

Treatment with SEM improved GSK3β levels in a murine model of PD, restoring them to levels comparable to those seen in wild-type animals. Additionally, phosphorylated GSK3β Y216 protein levels, which were elevated in untreated PD mice, returned to wild-type levels after SEM treatment (Table 1) [97].

### 6.3. CREB Gene Expression

Treatment with SEM elevated CREB mRNA levels in Pla2g6−/− mice, reinstating expression levels to those seen in wild-type controls [97].

### 6.4. Inflammation Response

Treatment with SEM markedly decreased CD68 immunoreactivity in all affected brain areas compared to controls [97].

Untreated mice displayed significantly heightened microglial activation compared to wild-type controls in various brain regions. SEM treatment notably decreased microglial activation, except in the cerebellum [97]. The study conducted by Zhang’s team [20,30,32] also revealed a decrease in microgliosis in the substantia nigra, thereby improving the chronic inflammation response in the brain. PD animals exhibited significant astrogliosis in various brain regions; SEM treatment alleviated astrogliosis, particularly in the hippocampus, with no statistically significant difference observed in the thalamus and cerebellum [97], but also in the substantia nigra [20,30,32]. SEM demonstrates greater efficacy in reducing astrogliosis and microgliosis compared to liraglutide [30,32,103].

### 6.5. Oxidative Stress

Both SEM and liraglutide reduce oxidative stress, as measured by 4-hydroxynonenal biomarker levels. SEM displays greater effectiveness in diminishing oxidative stress by inhibiting the formation of ROS [20,30,32,102]. Both SEM and liraglutide protect against the detrimental effects of 6-OHDA by suppressing the elevation of ROS levels within cells. While exposure to 6-OHDA resulted in a notable increase in ROS levels, neither SEM nor liraglutide alone affected ROS levels compared to the control group. However, when combined with 6-OHDA, both SEM and liraglutide effectively lowered ROS levels, with SEM exhibiting superior efficacy in reducing ROS levels compared to liraglutide (Table 1) [23].

### 6.6. Autophagy Regulation

The activation of Beclin-1 [20,23,32] initiates autophagosome formation, initiating the process of autophagy. The protein ATG7 is essential for lysosome formation and contributes to the delayed neurodegeneration of dopamine (DA) neurons. P62, a critical cargo adaptor for selective autophagy, plays a central role in clearing misfolded proteins. SEM enhances the expression of Beclin-1, ATG7, and LC3 (leading to increased conversion of LC3B-I to LC3B-II) [20,30,32], as well as P62. This suggests that the drug mitigates autophagy of dopaminergic neurons, potentially promoting neuroprotection [32].

### 6.7. Effects on Mitochondrial Membrane Potential (ΔΨm)

Both SEM and liraglutide counteract the damage inflicted by 6-OHDA on ΔΨm and mitochondrial dysfunction. Administration of either SEM or liraglutide alone does not impact ΔΨm levels [23]. However, when SEM or liraglutide is co-administered with 6-OHDA, there is a significant improvement in ΔΨm levels, indicating the prevention of 6-OHDA-induced mitochondrial toxicity. Notably, SEM demonstrates greater efficacy than liraglutide in enhancing ΔΨm levels [23].

## 7. SEM Effects on Motor Recovery

### 7.1. AD

#### 7.1.1. Open Field Test

There were no statistically significant differences observed in the total motor distance and the percentage of time spent in the central area among the four groups. The drug interventions did not influence the motor ability and spatial exploration ability of the mice [33,36].

#### 7.1.2. Novel Object Recognition Test

During the familiarization phase, no statistically significant differences were observed in the percentage of time spent exploring the first and second objects among the groups. However, in the testing phase, there was a significant difference in the novel object index (NOI) among the groups. The transgenic PD mice demonstrated a decreased NOI compared to WT mice (*p* < 0.01), and treatment with SEM increased NOI in PD mice (*p* < 0.05). Notably, this effect was reversed by the SIRT1 inhibitor EX527 [33,36].

#### 7.1.3. Y-Maze Task

No significant variance was observed in the total number of arm entries across the various groups. However, a notable distinction was evident in the percentage of correct spontaneous alternations among the groups. Notably, transgenic PD mice displayed a decrease compared to WT mice (*p* < 0.05), and treatment with SEM partially mitigated this decline (*p* < 0.05). It is noteworthy the protective effect was reversed by EX527 [33,36].

### 7.2. PD

#### 7.2.1. Rotarod Test

Pla2g6−/− mice untreated demonstrated significant progressive deficits from 10 to 14 weeks compared to age-matched wild-type controls [97]. However, Pla2g6−/− mice treated with 0.5 μg/g SEM did not display significant deficits in rotarod performance at any measured time point compared to wild-type controls [97]. MPTP impeded the mice’s ability to remain on the rotating rod for 3 min [32,103]. Post-hoc analysis revealed no difference between the control and SEM or liraglutide groups but a notable distinction between the control and MPTP groups. Both liraglutide and SEM ameliorated bradykinesia and imbalance induced by MPTP, with SEM showing greater efficacy [32,103].

#### 7.2.2. Vertical Pole Test

Commonly employed to evaluate locomotor function and hind limb strength, the vertical pole test revealed that untreated Pla2g6−/− mice exhibited notable progressive deficits beginning at 9 weeks. Administration of 0.5 μg/g SEM led to a marked enhancement in vertical pole test performance from weeks 9 to 13, although statistical significance was not sustained at 14 weeks [97].

#### 7.2.3. Open Field Test

Untreated Pla2g6−/− mice exhibited mobility parameter abnormalities at 12 weeks compared to the wild-type controls. Pla2g6−/− mice treated with high-dose SEM (0.5 μg/g—more than three times higher than the equivalent dose given to adult diabetics via subcutaneous administration) showed similar results to age-matched wild-type controls for most parameters, with notable differences observed in freezing time at 12 weeks [97]. Conversely, MPTP injection decreased spontaneous locomotion and overall travel time in mice [30,32]. Post-hoc analyses revealed no distinction between the control and liraglutide or SEM groups but a significant contrast between the control and MPTP groups [32]. SEM demonstrated greater efficacy than liraglutide in restoring MPTP-induced impairments in locomotor and exploratory activity [32]. In another study by Zhang et al. [103], there was an overall disparity in distance traveled, with no discrepancy between the control group and groups treated with liraglutide or SEM. However, significant variances were noted between the control and MPTP groups, MPTP + liraglutide and MPTP + SEM [103]. Both liraglutide and SEM normalized MPTP-induced impairments in locomotor and exploratory activity [103]. No significant distinction was observed between the MPTP + SEM and MPTP + liraglutide groups [103], despite SEM demonstrating superior benefits over liraglutide in the previous study [32].

#### 7.2.4. Footprint Test (Motor Coordination)

MPTP induced postural and gait abnormalities in mice, resulting in a significant overall difference between groups. Post-hoc analyses showed no distinction between the control and liraglutide or SEM groups but a notable difference between the control and MPTP groups. Both liraglutide and SEM ameliorated the abnormal posture and gait induced by MPTP, with SEM demonstrating greater effectiveness [32,103].

#### 7.2.5. Grip Strength Test

SEM and liraglutide both enhanced muscle strength impaired by MPTP, with SEM showing greater efficacy compared to liraglutide [103].

#### 7.2.6. Apomorphine-Induced Rotation

Variations in rotational behavior among the experimental conditions were evident, with significant differences observed across all groups [30]. The sham + saline group did not display contralateral rotations upon apomorphine challenge [30]. Both the 6-OHDA + saline group and the 6-OHDA + SEM group exhibited a notable increase in contralateral rotations following apomorphine injection compared to the sham + saline group (*p* < 0.0001) [30]. The 6-OHDA + SEM group showed a significant difference from the 6-OHDA + saline group (*p* < 0.001). Moreover, the 6-OHDA + DA5-CH group displayed significant differences from both the 6-OHDA + saline group (*p* < 0.001) and the 6-OHDA + SEM group (*p* < 0.0004) [30]. The study suggests that DA5-CH exhibits greater potency than SEM in safeguarding the brain against 6-OHDA toxicity [30].

## 8. Conclusions

In summary, the studies indicate a diverse potential for SEM in alleviating neurodegenerative mechanisms, offering valuable perspectives for its therapeutic use in associated conditions. The literature collectively presents compelling evidence regarding the neuroprotective capabilities of SEM in AD and PD. The drug mitigates AD pathology and associated symptoms, such as memory impairment, by fostering glycolysis through the GLP-1/SIRT1/GLUT4 pathway.

SEM proves effective in normalizing impaired motor function, preserving dopamine levels, reducing inflammation, oxidative stress, and apoptosis, and enhancing autophagy to safeguard against dopaminergic neuron loss in models of motor impairment.

Despite the promising preclinical results, further research is essential to fully elucidate the underlying mechanisms of SEM’s neuroprotective effects. Understanding these mechanisms is imperative for optimizing SEM’s therapeutic potential.

The ongoing clinical trials of SEM in AD and PD aim to provide crucial insights into SEM’s therapeutic potential, mechanisms of action, and optimal dosing strategies. Successful outcomes from these trials could lead to new, effective treatment options for millions of individuals affected by AD and PD, ultimately improving their quality of life and offering hope for better disease management.

## Figures and Tables

**Table 1:** Characteristics of animal studies that analyze the impact of SEM in PD.

Article	Animals/ Cells	Study Design (Randomization, Blind)	Pathology Model	Biochemical Tests	Behavior Tests	SEM (dose, Administration, Period of Time)	Other Drugs
Poupon-Bejuit 2022 [97]	Mice	Randomized, double-blind	Pla2g6−/− transgenic mice	- Hematoxylin/eosin staining - PAS staining. - Immunohistochemistry: CD68, NeuN, GFAP, Iba-1. - Western blot: RIP1, RIP3, β-actin. - PCR: GLP-1R gene, ATF-3, BCl2, BClxL Bax. - Stereology: pan-neuronal marker NeuN. - Blood analysis: total white blood cell count, neutrophils, lymphocytes, monocytes, eosinophils and basophils count, hematocrit, platelets, red blood cells, hemoglobin, and mean corpuscular volume.	Open field Rotarod Vertical pole	0.5, 0.25, 0.15 μg/g, i.p. injected once a week until the end of the experiment	No
Zhang 2018 [32]	Mice	Randomized	MPTP 20 mg/kg i.p. once daily for 7 days	- Immunohistochemistry: TH, GFAP, Iba-1, 4-HNE, BCl2, Bax. - Western blot: BCl2, Bax, β-actin, ATC7, LC2, Beclin1, SQSTM1, P62.	Open field Rotarod Footprint	25 nmol/kg, i.p., once daily for 7 days	Liraglutide 25 nmol/kg i.p. once daily for 7 days
Zhang 2019 [103]	Mice	In vivo, randomized	MPTP 20 mg/kg i.p. once daily for 30 days	- Immunohistochemistry: TH, GFAP, Iba-1, 4-HNE. - Western blot: BCl2, Bax, β-actin, α-Syn, ATC7, LC3, Beclin1, SQSTM1, GDNF.	Open field Rotarod Footprint gait Grip strength	25 nmol/kg, i.p., once daily for 30 days	Liraglutide 25 nmol/kg i.p. once daily for 30 days
Zhang 2022 [20]	Rats	Randomized, blind	6-OHDA	- ELISA: DA, TNF-α, IL-1β. - Western blot: TH, α-syn, IRS-1, IRS-1(phospho-S312), β-actin, GAPDH. - Immunofluorescence: TH.	Apomorphine Rotation	25 nmol/kg, i.p., once daily for 30 days postlesion	DA5-CH 25 nmol/kg, i.p., once daily for 30 days postlesion
Liu 2022 [23]	SH-SY5Y cells	In vitro	6-OHDA	- MTT method. - Western blot: LC3II, LC3I, Beclin1, P62, β-actin, ATG7. - Flow cytometry: measurement of ROS levels, membrane potential.	Not applicable	0, 1, 10, 100 nmol/L semaglutide	Liraglutide 0, 1, 10, 100 nmol/L

Abbreviations: Pla2g6−/−—Phospholipase A2, group VI knockout (a genetic modification where the Pla2g6 gene is knocked out), PAS—Periodic Acid-Schiff stain (a staining method used to detect glycogen and other carbohydrates), CD68—Cluster of differentiation 68 (a marker for macrophages and monocytes), NeuN—Neuronal Nuclei (a neuron-specific nuclear protein), GFAP—glial fibrillary acidic protein (a marker for astrocytes), Iba-1—Ionized calcium-binding adapter molecule 1 (a marker for microglia), RIP1—Receptor-interacting protein 1, RIP3—rRceptor-interacting protein 3, β-actin—Beta-actin (a cytoskeletal protein often used as a loading control in Western blots), PCR—Polymerase chain reaction, GLP-1R gene—Glucagon-like peptide-1 receptor gene, ATF-3—activating transcription Factor 3, BCl2—B-cell lymphoma 2 (anti-apoptotic protein), BClxL—B-cell lymphoma-extra large (anti-apoptotic protein), Bax—Bcl-2-associated X protein (pro-apoptotic protein), MPTP—1-Methyl-4-phenyl-1,2,3,6-tetrahydropyridine (a neurotoxin), TH—Tyrosine Hydroxylase (an enzyme involved in dopamine synthesis), 4-HNE—4-Hydroxynonenal (a marker of oxidative stress), ATC7—Autophagy-related gene 7 (part of the autophagy process), LC2—Light chain 2 (part of the autophagy process), Beclin1—Bcl-2-interacting myosin-like coiled-coil protein 1, SQSTM1—Sequestosome-1 (also known as P62, a protein involved in autophagy), P62—Sequestosome-1, α-Syn—Alpha-synuclein (a protein associated with neurodegenerative disorders), LC3—Microtubule-associated protein 1A/1B-light chain 3 (part of the autophagy process), GDNF—Glial cell-derived neurotrophic Factor, 6-OHDA—6-Hydroxydopamine (a neurotoxin), DA—Dopamine, TNF-α—Tumor necrosis factor-alpha, IL-1β—Interleukin-1 beta, IRS-1—Insulin receptor substrate 1, IRS-1(phospho-S312)—Phosphorylated form of IRS-1 at serine 312, GAPDH—Glyceraldehyde-3-phosphate dehydrogenase (a common housekeeping gene used as a loading control in experiments), DA5-CH—Dopamine-5,6-chlorohydroxydihydroxyphenylalanine, ATG7—Autophagy-related gene 7, LC3II—Microtubule-associated protein 1A/1B-light chain 3 (processed form), LC3I—Microtubule-associated protein 1A/1B-light chain 3 isoform I, ROS—Reactive oxygen species.
